# Identification of K_Ca_3.1 Channel as a Novel Regulator of Oxidative Phosphorylation in a Subset of Pancreatic Carcinoma Cell Lines

**DOI:** 10.1371/journal.pone.0160658

**Published:** 2016-08-05

**Authors:** Ilya Kovalenko, Andrea Glasauer, Laura Schöckel, Daniel R. P. Sauter, Alexander Ehrmann, Florian Sohler, Andrea Hägebarth, Ivana Novak, Sven Christian

**Affiliations:** 1 Drug Discovery, Therapeutic Research Groups / Onc II, Bayer Pharma AG, Berlin, Germany; 2 Department of Bioanalytics, Institute of Biotechnology, Technische Universität Berlin, Berlin, Germany; 3 Section for Cell Biology and Physiology, Department of Biology, University of Copenhagen, Copenhagen, Denmark; 4 Drug Discovery, Lead Discovery / Cell Biology, Bayer Pharma AG, Berlin, Germany; 5 Drug Discovery, Therapeutic Research Groups / Bioinformatics, Bayer Pharma AG, Leverkusen, Germany; National Institute of Environmental Health Sciences, UNITED STATES

## Abstract

Pancreatic ductal adenocarcinoma (PDAC) represents the most common form of pancreatic cancer with rising incidence in developing countries and overall 5-year survival rates of less than 5%. The most frequent mutations in PDAC are gain-of-function mutations in *KRAS* as well as loss-of-function mutations in *p53*. Both mutations have severe impacts on the metabolism of tumor cells. Many of these metabolic changes are mediated by transporters or channels that regulate the exchange of metabolites and ions between the intracellular compartment and the tumor microenvironment. In the study presented here, our goal was to identify novel transporters or channels that regulate oxidative phosphorylation (OxPhos) in PDAC in order to characterize novel potential drug targets for the treatment of these cancers. We set up a Seahorse Analyzer XF based siRNA screen and identified previously described as well as novel regulators of OxPhos. The siRNA that resulted in the greatest change in cellular oxygen consumption was targeting the *KCNN4* gene, which encodes for the Ca^2+^-sensitive K^+^ channel K_Ca_3.1. This channel has not previously been reported to regulate OxPhos. Knock-down experiments as well as the use of a small molecule inhibitor confirmed its role in regulating oxygen consumption, ATP production and cellular proliferation. Furthermore, PDAC cell lines sensitive to K_Ca_3.1 inhibition were shown to express the channel protein in the plasma membrane as well as in the mitochondria. These differences in the localization of K_Ca_3.1 channels as well as differences in the regulation of cellular metabolism might offer opportunities for targeted therapy in subsets of PDAC.

## Introduction

Pancreatic ductal adenocarcinoma (PDAC) represents the most common form of pancreatic cancer with increasing incidence in developing countries. It is an aggressive and highly metastatic cancer with an overall 5-year survival rate of less than 5% [1]. Inactivation of the *p53* tumor suppressor gene and mutationally activated *KRAS* oncogene are the most common alterations in PDAC. Mutations in *KRAS* are present in >90% of PDAC and are the earliest genetic alterations [2], [3]. The chemotherapeutic gemcitabine is the first-line standard of care as it was shown to increase the median overall survival from 4.41 to 5.65 months [4], [5]. However, most clinical trials combining gemcitabine with other targeted therapies have failed or showed only a minor therapeutic benefit. Therefore, there is an urgent need to identify alternative drug targets for the treatment of PDAC.

It is widely recognized that cancer cells adapt their metabolic pathways during transformation to gain a survival advantage [6]. Predominantly, many tumor cells are characterized by aerobic glycolysis [7], which entails a high rate of glucose uptake and subsequent activity of glucose transporters (GLUTs) [8], as well as a high excretion rate of lactate, even in the presence of oxygen [9]. Consequently, many metabolic enzymes and transporters are regulated by oncogenes and/or tumor suppressor genes. *p53* [10] upregulates the expression of GLUTs, TP53-inducible glycolysis and apoptosis regulator (TIGAR), [11], [12] and mitochondrial respiration [13], [14], [15]. In contrast, lack of oxygen or adequate nutrients upregulates *p53* [16], [17], [18]. In PDAC cells *KRAS* mutations [19] were shown to modulate expression of hexokinase 2, which shuttles glucose towards glycolysis and lactate production [20]. Furthermore, PDAC cells display an increased uptake of glutamine, which is transported to mitochondria where it is converted to aspartate. Aspartate is transported to the cytosol where it is transaminated into oxaloacetate by glutamic-oxaloacetic transaminase 1 (*GOT-1*) and enters the TCA cycle to produce reducing equivalents by increasing the NADPH/ NADP^+^ ratio [21]. Additionally, oncogenic *KRAS* was shown to increase nuclear factor (erythroid-derived 2)-like 2 (*NRF2*) expression which further protects cancer cells from reactive oxygen species (ROS)-induced toxicity [22]. Overall, it is evident that the two most common mutations in PDAC, inactivating *p53* and activating *KRAS*, lead to profound metabolic changes, i.e., increase in glycolysis, glutaminolysis, and decrease in mitochondrial activity and oxygen consumption.

Many of the described metabolic pathways depend on transporters to supply the cell with nutrients such as glucose and amino acids and to export waste products such as lactate [23]. Furthermore, ion channels influence many physiological parameters of the cell, including metabolic pathways [24]. Screening approaches directed toward the transportome genes were successfully used previously to identify transporters mediating chemoresistance in the panel of NCI-60 cells and the role of the transportome in tumor angiogenesis was recently reviewed [25]. Therefore, we aimed to identify novel transporters or channels that regulate the metabolism of pancreatic tumor cells and can be potentially exploited as drug targets for the treatment of PDAC. For this purpose, we established a Seahorse XF Analyzer based siRNA screen to identify transporters and/or ion channels regulating OxPhos in the PDAC cell line Mia PaCa-2. In our screen, we identified the Ca^2+^-sensitive channel K_Ca_3.1 encoded by the *KCNN4* gene as a novel regulator of oxygen consumption in a subset of PDAC cells, further characterized mitochondrial expression of K_Ca_3.1 isoform and observed it to at least partially contribute to the observed effects on oxygen consumption in these cells.

## Materials and Methods

### Cell lines and compounds

Panc-1 cells were cultured in DMEM with 10% fetal calf serum; AsPC-1 and BxPC-3 cells were cultured in RPMI 1640 with 10% fetal calf serum, Capan-1 cells were cultured in IMDM with 20% fetal calf serum; Mia PaCa-2 cells were cultured in DMEM/F12 with 10% fetal calf serum and 2.5% horse serum; All media used for routine passaging contained GlutaMAX and 25 mM glucose. KCNN4 inhibitor rac-16 was synthesized as described before [26], NS309 and TRAM-34 were obtained from Sigma-Aldrich (St. Louis, MO, USA), diluted in DMSO to 10 mM stock solutions and stored in -20°C. Oligomycin (Oligo), FCCP (carbonyl cyanide-4 (trifluoromethoxy) phenylhydrazone), rotenone (R) and antimycin A (A) were obtained from Sigma-Aldrich (St. Louis, MO, USA), diluted in DMSO to 10 mM stock solutions and stored in -20°C. Saponin, TMPD (N,N,N′,N′-tetramethyl-p-phenylenediamine), and sodium ascorbate were obtained from Sigma-Aldrich, (St. Louis, MO, USA).

### Metabolic Flux analysis

For Metabolic Flux analysis the Seahorse XF96 instrument (Seahorse Bioscience, Chicopee, MA, USA) was used and all the procedures were performed according to manufacturer’s instructions. Briefly, 25 000 cell/well were seeded the night before performing the assay. Cells were washed twice with Assay medium containing 11 mM glucose, 2 mM sodium pyruvate and 2 mM glutamine and pre-incubated for 1 hr in 37°C in a non-CO_2_ incubator. Assay was performed with 4 min mixing, followed by 4 min of measurement.

### Seahorse siRNA Screen

siRNAs were selected from the Ambion Silencer Select Human Extended Druggable Genome siRNA library (Life Technologies, Carlsbad, CA, USA) which consisted of pooled siRNAs of 3 differrent sequences per target gene. Hiperfect transfection reagent (Qiagen, Hilden, Germany) was used according to manufacturer’s manual, briefly, siRNA/Hiperfect complexes were prepared in 10 μl OptiMEM to have a final concentration of 10 nM of siRNA and 0.5 μl Hiperfect per well. 2000 cells/well of Mia PaCa-2 cells were seeded in 10 μl of growth media into a Seahorse microplate, incubated for 4 hrs with transfection particles, and 180 μl of growth media was added. 6 independent wells per each gene were transfected, 3 wells for siRNA targeting SLC2A1 (Glut-1), 3 wells for siRNA targeting UCP-2 and 3 wells for siRNA targeting PLK-1 as well as 6 wells of untransfected controls were included in each screening plate. Transfected cells were incubated 72 hrs prior to Seahorse Metabolic flux Analysis. Cells were washed two times with assay medium, 200 μl of assay medium supplied with a 1:1000 dilution of Hoechst dye (Sigma-Aldrich, St. Louis, MO, USA) and incubated for 1 hr in a non-CO_2_ incubator. After the samples were analyzed, the Hoechst stained nuclei were imaged with the ImageXpress ® Micro XLS System and MetaMorph® software was used to assay the cell number (Molecular Devices, Sunnyvale, CA, USA).

For the analysis of the screening data, the average oxygen consumption rate (OCR) and extracellular acidification rate (ECAR) of 4 measurements for 6 wells was taken, the nuclei count was performed from the images of 8 squares per well and averaged, the average of 6 wells then was taken for further Cell number adjustments of OCR and ECAR values. Each plate contained si-PLK-1 (Polo-like kinase 1) control of transfection efficiency, 4 pooled si-SLC2A1 (Glut-1) and si-UCP-2 (Mitochondrial uncoupling protein 2) for Metabolic Flux analysis control, together with scrambled siRNA (Dharmacon, Lafayette, CO, USA) as well as untransfected controls. The results were calculated as relative to the average of the entire 96-well plate (except controls) to remove any inter-plate bias. Hits were considered if cell-number adjusted OCR differed more than 10% from the plate average.

Knock-down confirmation on mRNA level after the screening procedure was not performed due to the fact that most of the samples were fixed in formalin for the subsequent nuclei count.

### XF Cell Mito Stress test

The protocol was according to supplier manual (Seahorse Bioscience, Chicopee, MA, USA) with the following adaptations: 20 000 cell/well were seeded the night before the assay. Cells were washed twice with Assay medium containing 11 mM glucose, 2 mM sodium pyruvate and 2 mM glutamine and pre-incubated for 1 hr in 37°C in a non-CO_2_ incubator. Metabolic Flux assay at the Seahorse XF96 instrument was performed with 4 min mixing, followed by 4 min of measurement, after initial baseline measurement cells were treated with rac-16, following 4 cycles of recordings, 1 μM of oligomycin final concentration was injected to each well and after mixing 4 cycles of mixing and recording were performed. 0.5 μM of FCCP final concentration was injected following 4 cycles of measurements of maximal respiration. The mix of 1 μM of antimycin A and 1 μM of rotenone final concentrations was injected finally and 4 measurement cycles were performed.

### Oxygen consumption measurement in permeabilized cells

Mannitol and Sucrose (MAS) buffer was prepared fresh in distilled, deionized water containing 70 mM sucrose, 220 mM mannitol, 10 mM KH_2_PO_4_, 5 mM MgCl_2_, 2 mM HEPES and 1 mM EGTA. The buffer was pH adjusted to 7.2 with 0.1 M KOH and filter-sterilized. Cell permeabilization was performed as described before [27]. Briefly, Mia PaCa-2 cells were seeded, incubated for 48 hrs, shortly before the assay growth media was substituted with MAS buffer and analyzed by Seahorse XF96 instrument as described. 25 μg/ml Saponin, 0.5 mM TMPD and 2 mM ascorbate were injected after 17 min of 3 baseline measurements, followed by the addition of 10 μM rac-16 as indicated after 24 min of permeabilized cells respiration measurements. For respiration measurements of permeabilized si-NT or si-KCNN4 Mia PaCa-2 cells, cells were transfected as described below. Seahorse measurements were carried out 24h after the second transfection. 30 000 cell/well were seeded the night before the assay.

### Screening confirmation protocol

For Screening hits confirmation experiments 4x10^5^ Mia PaCa-2 cells were seeded in each well of 6 well plate and reverse-transfected according to Lipofectamine RNAiMAX (Life Technologies, Carlsbad, CA, USA) protocol, for KCNN4 the following siRNAs were ordered from Dharmacon (Lafayette, CO, USA):

KCNN4 si1: CAUCGGCGCUCUCAAUCAA

KCNN4 si2: ACAAGAAGCCUGGAUGUUC

KCNN4 si3: CCGAGAGGCAGGCUGUUAA

KCNN4 si4: GCACUGGAGUCAUGGGUGU

Cells were split after 2 days for RNA (48 hrs post-transfection) extraction and seeded for re-transfection with corresponding siRNAs. Protein samples were prepared 5 days post transfection.

### Gene expression analysis

For the gene expression analysis the cells were harvested and processed according to the NucleoSpin RNA Mini (Macherey-Nagel, Düren, Germany) protocol using QiaCube System (Qiagen, Hilden, Germany) in accordance with the manufacturer’s manual. RNA concentration was determined using NanoDrop. 1 μg of total RNA was used for cDNA synthesis according to Maxima Reverse Transcriptase protocol (Life Technologies, Carlsbad, CA, USA). 20 μl reactions were incubated in Applied Biosystems Veriti® 96-Well Thermal Cycler for 10 min at 25°C, 30 min at 50°C, 5 min at 95°C, and then incubated at 4°C.

Real-time PCR was performed using an Applied Biosystems Step-One Real-Time PCR System. The 20 μl PCR included 0.5 μl RT product, 1× TaqMan® Fast Advanced Master Mix and 1 μl of TaqMan® Gene Expression Assay (Applied Biosystems, Foster City, CA, USA). Expression Assay, Life Technologies Ordering information: Assay ID: Cf02696785_m1, Gene Aliases: *KCNN4*; Catalog # 4351372 and 1 μl of endogenous control. For the normalization purposes Human HPRT1 (HGPRT) Endogenous Control (VIC® ⁄ MGB Probe, Primer Limited) (Applied Biosystems, Foster City, CA, USA) was used in a multiplexed reaction together with FAM-labeled KCNN4 probe. The reactions were incubated in a 96-well optical plate at 95°C for 10 min, followed by 40 cycles of 95°C for 15 sec and 60° for 10 sec. The Ct data was determinate using default threshold settings. The threshold cycle (Ct) is defined as the fractional cycle number at which the fluorescence passes the fixed threshold.

### Western Blot

Anti-KCNN4 (final concentration 1 μg/ml) and anti-β actin (1:5000 dilution) antibody were obtained from Sigma-Aldrich, St. Louis, MO, USA. Total OXPHOS Human WB Antibody Cocktail (final concentration 3 μg/ml) was obtained from Abcam, Cambridge, UK; IRDye® 800CW Goat anti-Rabbit IgG (H + L), IRDye® 800CW Goat anti-Mouse IgG2a were obtained from LI-COR Biosciences Lincoln, NE, USA. Secondary antibodies were used at 0.2 μg/ml final concentration.

Briefly, cells were harvested in RIPA buffer (Life Technologies, Carlsbad, CA, USA) supplied with protease inhibitors, complete Mini EDTA-free (Roche Life Sciences, Indianapolis, IN, USA) and phosphatase inhibitors PhosphoSTOP (Roche Life Sciences, Indianapolis, IN USA). Lysate was centrifuged for 15 min at 4°C, protein concentration was determined by a BCA Protein Assay (Life Technologies, Carlsbad, CA, USA). Protein samples were reduced and denaturated in 4X NuPAGE® LDS Sample Buffer and 10X NuPAGE® Sample Reducing Agent (Life Technologies, Carlsbad, CA, USA) by incubating for 5 min at 80°C. Proteins were separated on 4–12% Bis-Tris NuPAGE gels and dry-transferred to nitrocellulose membranes and blocked in 5% non-fat milk-TBST. The membranes were incubated with antibodies at 4°C overnight and after thorough washing, the membranes were incubated with the corresponding secondary antibody for 1 hr. Finally, the membranes were thoroughly washed and subjected to imaging by ODYSSEY® CLx (LI-COR Biosciences, Lincoln, NE, USA).

### Mitochondria Isolation

The Mitochondria Isolation Kit for Cultured Cells (Life Technologies, Carlsbad, CA, USA) was used to isolate mitochondria according to manufacturer’s instructions, the Dounce homogenization protocol was performed with 60 strokes. The supernatant was taken as the cytosolic fraction. Mitochondrial pellets were resuspended in 1% CHAPS buffer for further Western Blot processing.

### Proliferation assay

Cellular growth kinetics were analyzed using the xCELLigence RTCA MP Station (ACEA Biosciences, San Diego, CA, USA) according to manufacturer’s instructions. For the galactose treatment, cells were resuspended in galactose media and seeded in xCELLigence plates. Rac-16 treatment was performed as indicated and added to the cells 48 hrs after seeding in glucose or galactose-containing media. The slope of the exponential phase of the growth curve was calculated in the complementary software. The Relative Growth (%) was calculated as the ratio between the slope of DMSO-treated cells and the rac-16-treated cells in the appropriate media.

### Cell viabilty assay

Cells were seeded in triplicates in 96-well plates at a density of 2000 cells/well. 72 hours after the second siRNA transfection cell viability was measured using CellTiter-Glo (CTG, Promega) according to the manufacturer´s protocol. Luminescence was detected in a microplate reader (Tecan) after 15 min of incubation.

### Electrophysiology

Whole-cell patch-clamp recordings were performed using the Axopatch 200B amplifier interfaced to a Digidata 1440A controlled by pClamp10 software (Molecular Devices, Sunnyvale, CA, USA). Analog signals were acquired at 2.5 kHz and filtered at 1 kHz. Glass electrodes were prepared from borosilicate glass and had an input resistance of 2–6 MΩ when filled with pipet solution contained (in millimolar): 140 K-Aspartate, 8 Na-Aspartate, 4.3 CaCl_2_, 2.06 MgCl_2_, 5 K-EGTA, 10 HEPES, pH 7.2, free Ca^2+^ was 1 μM, calculated by MaxChelator software (Stanford University). TRAM-34 concentration was 1 μM. The standard bath solution contained (in millimolar): 150 Na-Aspartate, 5 KCl, 2 CaCl_2_, 1 MgCl_2_, 10 Glucose, 10 HEPES, pH7.4. All recordings were performed at room temperature (20°C).

### ATP production measurement

Mia PaCa-2 cells were incubated in DMEM with 25 mM galactose overnight, followed by compound treatment for 4 hrs. Finally, cells were subjected to ATP measurement using CellTiter Glo assay (Promega, Madison, WI, USA) according to manufacturer’s instructions. 1 μM of Oligomycin (Sigma-Aldrich, St. Louis, MO, USA) was used as positive control.

### CellTiter Blue assay

Mia PaCa-2 cells were seeded in a Corning 96 well tissue culture plate washed 2 times with PBS containing Ca^2+^/Mg^2+^ and incubated for 2 hrs in the Assay media supplied with different carbon sources as described. CellTiter-Blue Cell Viability Assay (Promega, Madison, WI, USA) was used according to the manufacturer’s instructions.

### Statistical analysis

All the samples contained at least 6 replicates. Statistical analysis was performed using GraphPad Prism software version 6.0 (GraphPad Software, La Jolla, CA, USA) for ANOVA test. Statistical significance is indicated ns–not significant, p-value is >0.05, * for p< 0.05 and ** for p<0.01; Standard Deviation was calculated in Microsoft Excel. When comparing different conditions or expression in the panel of cell lines, ANOVA Tukey test was used and the datasets were considered significantly different when p<0.05. For the effects of siRNA or compound treatments ANOVA test with Bonferroni post-hoc and Dunnett’s two-tailed t-test was used as described in the figure legends.

## Results

### Screening conditions for the identification of novel metabolic regulators

**Fig 1:**
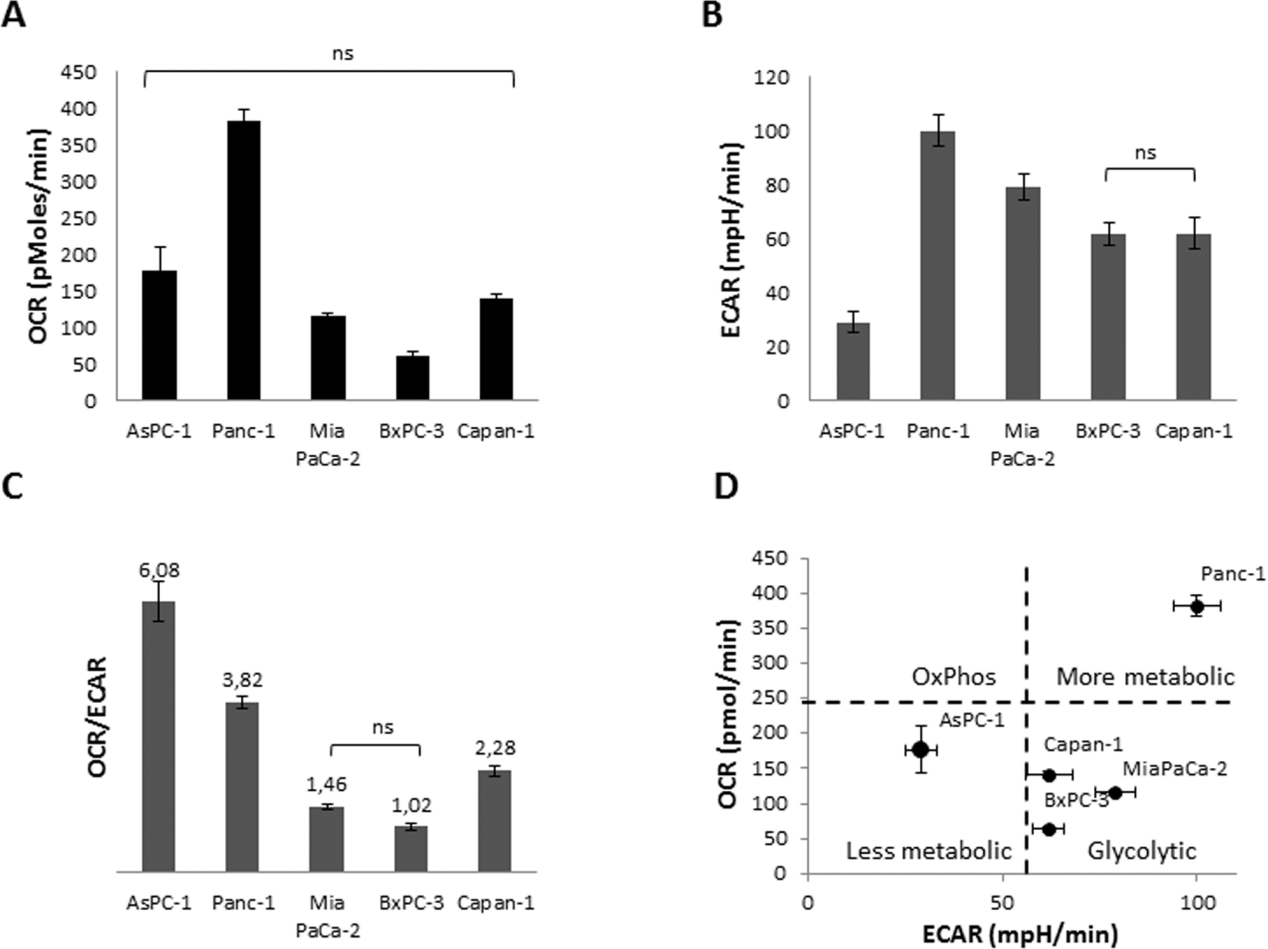
Metabolism of PDAC cell lines. **(A)** Measurement of OCR, **(B)** ECAR, **(C)** OCR/ECAR and **(D)** metabolic phenotyping were performed in Seahorse assay medium supplied with 11 mM Glucose, 2 mM Pyruvate and 2 mM Glutamine. 20 000 cells/well were seeded one day prior to the experiment. n = 12 for each cell line. Tukey’s Multiple Comparison ANOVA test was performed. All groups are significantly different unless indicated otherwise.

Five different PDAC cell lines were characterized for the metabolic phenotype by measuring oxygen consumption rate (OCR) (Fig 1A) and extracellular acidification rate (ECAR) as an indicator of glycolysis, respectively, (Fig 1B) using the Seahorse XF Analyzer. OCR is an indicator of mitochondrial respiration, and ECAR (proton excretion) is largely the result of glycolysis (http://www.seahorsebio.com/). All five cell lines showed different metabolic characteristics with Panc-1 cells being metabolically most active, as indicated by high ECAR and OCR. AsPC-1 cells were characterized by a low glycolytic rate, whereas BxPC-3 and Mia PaCa-2 cells were the most glycolytic (Fig 1C and 1D). As pancreatic tumors are known to be rather glycolytic, we selected Mia PaCa-2 cells for further establishment of optimal conditions for a siRNA-based screening approach to identify novel carrier or channel proteins that influence OxPhos, to optimize transfection conditions as well as optimal assay conditions in the Seahorse XF Analyzer.

**Fig 2:**
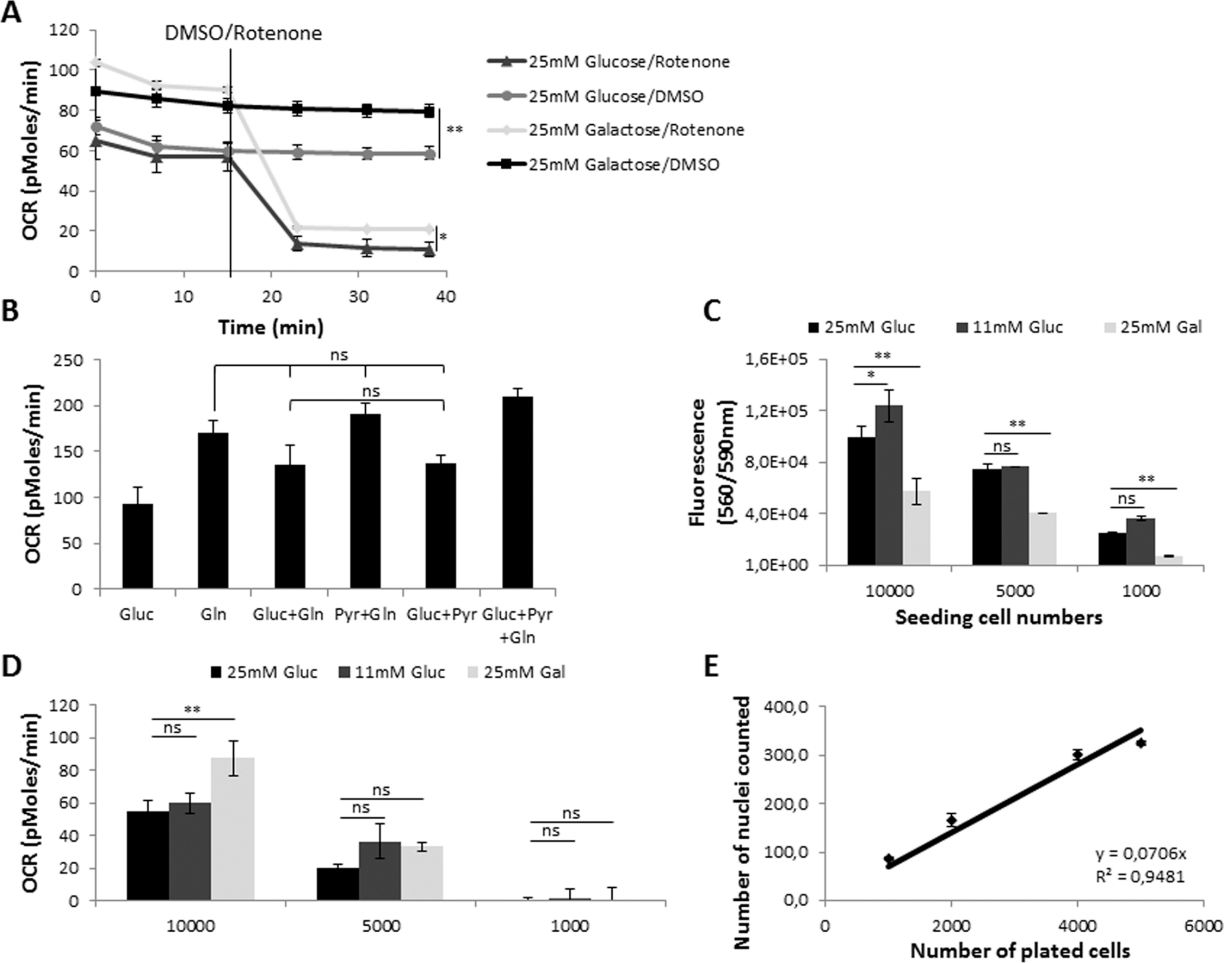
Optimization of screening conditions in Mia PaCa-2 cells. **(A)** Oxygen consumption measurements (OCR) of Mia PaCa-2 cells in 25mM glucose or 25mM galactose. The Complex I inhibitor rotenone (1 μM) was used as a positive control for the inhibition of OxPhos, n = 6; **(B)** Measurement of OCR in media supplied with different carbon sources: 25 mM Glucose, 2 mM Glutamine, 2 mM Na Pyruvate, n = 12; **(C)** CellTiter Blue fluorescence measurements of cell titrations cultured in different assay media for 2 hrs, n = 12; **(D)** OCR measurements of Mia PaCa-2 cell titrations in different assay media, n = 12; **(E)** Nuclei number count using Hoechst stain in Seahorse plate, n = 12. For A, t-test was performed, *, p-value is 0.01 to 0.05; **, p-value is 0.001 to 0.01; B, Tukey’s Multiple Comparison ANOVA Test was performed. Groups are significantly different unless indicated otherwise. For C and D, two-tailed t-test was performed, ns, not significant, p>0.05, *, p-value is 0.01 to 0.05; **, p-value is < 0.01.

The influence of different carbon sources on cellular metabolism was also determined [28]. As expected, replacing glucose with galactose led to a switch from glycolysis to OxPhos, as indicated by elevated oxygen consumption (Fig 2A). Replacing glucose by 2 mM glutamine led to an increased rate of oxygen consumption attributable to the glutaminolysis, addition of 2 mM pyruvate or glucose did not significantly increase respiration, Addition of either pyruvate or glutamine to glucose increased respiration to equal extent.[29]. The highest OCR was observed by combining all three carbon sources that feed into the TCA cycle and thereby fuel respiration, therefore we determined the optimal screening conditions to be 10 mM glucose, 2 mM glutamine and 2 mM pyruvate (Fig 2B). For screening purposes, cell number titration experiments were pursued to determine the optimal cell number that allowed efficient transfection as well as determination of oxygen consumption rate. Titration of the cell number revealed a minimum of 5000 tumor cells per well to reliably detect OCR independent of the carbon source (Fig 2C). Changes in oxygen consumption were normalized to cell number. Determining the cell number by counting Hoechst stained nuclei allowed detection of changes independent of the metabolic phenotype (Fig 2D and 2E).

To identify novel carrier or channel proteins regulating mitochondrial metabolism in PDAC cell lines, genes were selected based on gene ontologies (GO) associated with transport or carrier. 18 GOs were selected which led to the identification of 786 putative transporters or carriers. To determine which of these genes are expressed in Mia PaCa-2 cells, we utilized in-house microarray gene expression data based on the analysis of 14 biological replicates with Affymetrix Gene expression arrays. We used the Affymetrix detection call to determine if a gene was expressed. The fraction of samples with a positive Affymetrix detection call was averaged across all probe sets of a gene, and the gene was considered to be expressed if this value exceeded 0.4. All the transportome genes identified as being expressed in Mia PaCa-2 cells were compared with the available Ambion Silencer Select Human Extended Druggable Genome siRNA library which led to the identification 223 transportome genes (S1 Table).

### K_Ca_3.1 channels regulate oxygen consumption specifically in a subset of pancreatic tumor cell lines

The siRNA based metabolic screening approach of 223 transportome genes in Mia PaCa-2 cells maintained in 10 mM glucose, 2 mM glutamine and 2 mM pyruvate led to the identification of 13 hits with an inhibition of cell number adjusted OCR ≥ 10% (Table 1) and 15 hits with an increase of cell number adjusted OCR ≥ 10% (Table 2). These included known regulators of metabolism, such as *SLC2A3*, *SLC2A1* and *BSG*, as well as novel regulators previously not associated with tumor cell metabolism.

**Table 1:** Transportome hits which downregulate oxygen consumption >10%.

Gene/ Gene ID	Inhibition OCR CA, %	Cell Index, %	Affymetrix expression	Name and function
KCNN43783	22	93	1.00	Intermediate conductance calcium-activated potassium channel protein 4, K_Ca_3.1
SLC26A21836	21	94	1.00	Sulfate transporter.
NEDD4L23327	20	95	1.00	E3 ubiquitin-protein ligase NEDD4-like
SLC19A210560	20	84	1.00	Thiamine transporter 1
PSEN15663	17	72	1.00	Presenilin-1; L-glutamate transport; gamma-secretase complex, an endoprotease complex that catalyzes the intramembrane cleavage of integral membrane proteins such as Notch receptors and APP (beta-amyloid precursor protein)
SLC2A14144195	17	94	1.00	GLUT14, GLUT3 duplication. Solute carrier family 2, facilitated glucose transporter member 14
KCNT2343450	16	96	0.43	Potassium channel subfamily T member 2
SLC25A3281034	16	129	1.00	Mitochondrial folate transporter/carrier; Transports folate across the inner membranes of mitochondria.
SLC25A123560	15	102	1.00	Tricarboxylate transport protein, mitochondrial. Involved in citrate-H+/malate exchange. Important for the bioenergetics of hepatic cells as it provides a carbon source for fatty acid and sterol biosyntheses, and NAD+ for the glycolytic pathway.
SCO29997	13	106	1.00	Acts as a copper chaperone, transporting copper to the Cu(A) site on the cytochrome c oxidase subunit II (COX2), complex IV assembly
SLC12A26558	12	93	1.00	Basolateral Na-K-Cl symporter
SLC1A46509	11	97	0.93	ASCT1 protein, Neutral amino acid transporter A; Transporter for alanine, serine, cysteine, and threonine. Exhibits sodium dependence.
BSG682	10	82	1.00	Basigin; Major regulator of SLC16A1, SLC16A3 and SLC16A8 activity

**OCR:** oxygen consumption rate; **CA:** cell number adjusted; **Cell index:** Changes in cell number compared to control transfected cells.

**Table 2:** Transportome hits which upregulate oxygen consumption >10%.

Gene/ Gene ID	Induction OCR CA, %	Cell Index, %	Affymetrix expression	Name and function
RASA322821	10	106	0.57	Ras GTPase-activating protein 3; calcium-release channel activity
ABCD45826	11	84	1.00	May be involved in intracellular processing of vitamin B12 (cobalamin). Could play a role in the lysosomal release of vitamin B12 into the cytoplasm.
TAP16890	11	84	0.71	Antigen peptide transporter
TPCN2219931	12	114	1.00	Two pore calcium channel protein 2; Nicotinic acid adenine dinucleotide phosphate (NAADP) receptor that may function as one of the major voltage-gated Ca2+ channels (VDCC) across the lysosomal membrane
TNPO13842	13	90	1.00	Transponin, nuclear transport of proteins
SLC25A168034	14	118	1.00	Graves disease autoantigen; Required for the accumulation of coenzyme A in the mitochondrial matrix
SLC7A823428	15	73	0.64	LAT2, Large neutral amino acids transporter small subunit 2
SLC2A36515	16	97	1.00	GLUT3; Solute carrier family 2, facilitated glucose transporter member 3
SLC25A1310165	17	114	1.00	Calcium-binding mitochondrial carrier protein Aralar2; Catalyzes the calcium-dependent exchange of cytoplasmic glutamate with mitochondrial aspartate across the mitochondrial inner membrane. May have a function in the urea cycle.
SLC12A710723	22	127	1.00	Potassium-chloride cotransporter 4
ITGAV3685	23	57	1.00	Integrin 5-alpha
SLC2A16513	27	68	1.00	Glut-1, Solute carrier family 2, facilitated glucose transporter member 1
NUDT953343	28	110	1.00	ADP-ribose pyrophosphatase, mitochondrial
CUL58065	31	108	1.00	Vasopressin-activated calcium-mobilizing receptor 1; p53 regulated,
SLC16A26567	41	99	0.79	MCT8, Monocarboxylate transporter 8. Very active and specific thyroid hormone transporter. Stimulates cellular uptake of thyroxine (T4), triiodothyronine (T3), reverse triiodothyronine (rT3) and diidothyronine. Does not transport Leu, Phe, Trp or Tyr

**OCR:** oxygen consumption rate; **CA:** cell number adjusted; **Cell index:** Changes in cell number compared to control transfected cells.

**Fig 3:**
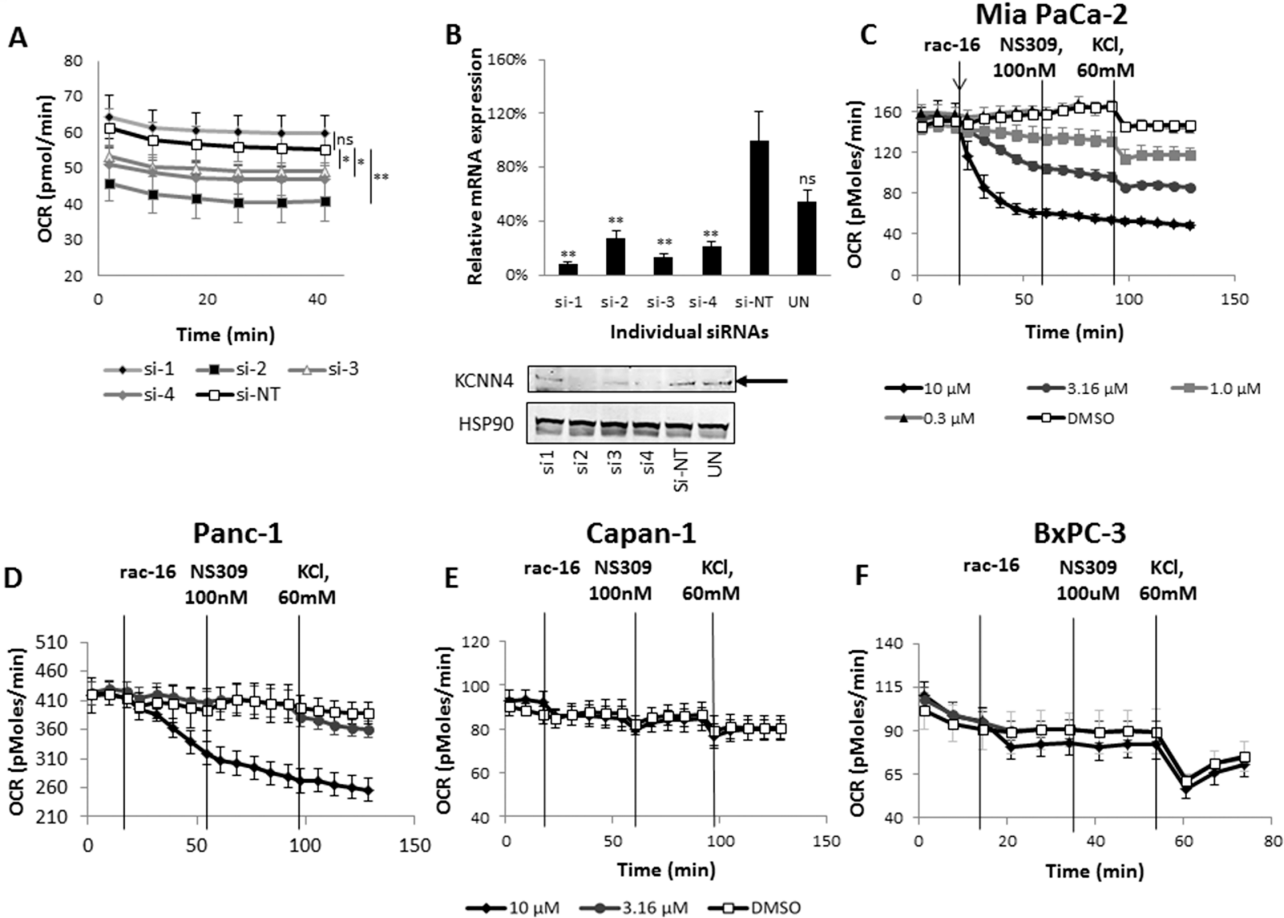
KCNN4 hit confirmation. **(A)** OCR of Mia PaCa-2 cells transfected with individual siRNAs against KCNN4, 20 000 cells/well were re-seeded the night before the assay to avoid cell number-dependent changes. n = 6; **(B)** Upper panel: *KCNN4* TaqMan gene expression assay. *KCNN4* mRNA levels were normalized to HPRT. K_Ca_3.1 Lower panel: Western Blot analysis of *KCNN4* protein expression, *HSP90* was used as loading control. Arrow indicates the KCNN4 protein band. **(C)** Oxygen consumption measurements of Mia PaCa-2, **(D)** Panc-1, **(E)** Capan-1 and **(F)** BxPC-3 cells treated with rac-16 (KCNN4 inhibitor) at different concentrations as indicated, 100 nM NS309 (KCNN4 activator) and high concentrations of extracellular KCl (60 mM), n = 5. Data are represented as the mean ± SD. ANOVA test with Bonferroni post-hoc was performed to compare NT siRNA and siRNA targeting KCNN4 mRNA; ns, not significant, p>0.05, *, p-value is 0.01 to 0.05; **, p-value is < 0.01.

Among these, the *KCNN4* gene was the number one hit that decreased cell number adjusted OCR by more than 20%. In order to confirm *KCNN4* as a hit regulating oxygen consumption in Mia PaCa-2 cells, four independent individual siRNAs were used; three out of four siRNAs led to a significant decrease of oxygen consumption (Fig 3A). All four siRNAs led to a significant reduction of K_Ca_3.1 channel mRNA (Fig 3B, upper panel). However, only siRNA 2, 3 and 4 led to a significant reduction on protein level, which is consistent with their effect on oxygen consumption (Fig 3B, lower panel). To further confirm the specificity of the OCR reduction, results were validated using the previously described K_Ca_3.1 channel inhibitor rac-16 [26], which resulted in a dose-dependent decrease of OCR in Mia PaCa-2 cells (Fig 3C). This effect could not be reversed by application of the K_Ca_3.1 channel-specific activator, NS309. Consistently, increasing the extracellular potassium concentration to 60 mmol/l also reduced OCR, an effect that decreased with increasing compound concentrations, further supporting that K_Ca_3.1 channel function mediates the observed decrease in OCR (Fig 3C). Interestingly, rac-16 (10 μM) had no effect on OCR of Capan-1, and only minor effects on BxPC-3 cells were observed. Panc-1 cells were only responsive at the highest concentration of 10 μM. These differences in OCR changes upon rac-16 treatment indicate a cell type-specific effect of K_Ca_3.1 channel inhibition on oxygen consumption (Fig 3D–3F).

Corresponding effects were observed on the extracellular acidification rate (ECAR) (Figure A in S1 File). Inhibition of K_Ca_3.1 channel by rac-16 led to a dose-dependent increase in ECAR in Mia PaCa-2 cells. Rac-16 had only a weak or no effect on ECAR in the other three PDAC cell lines.

**Fig 4:**
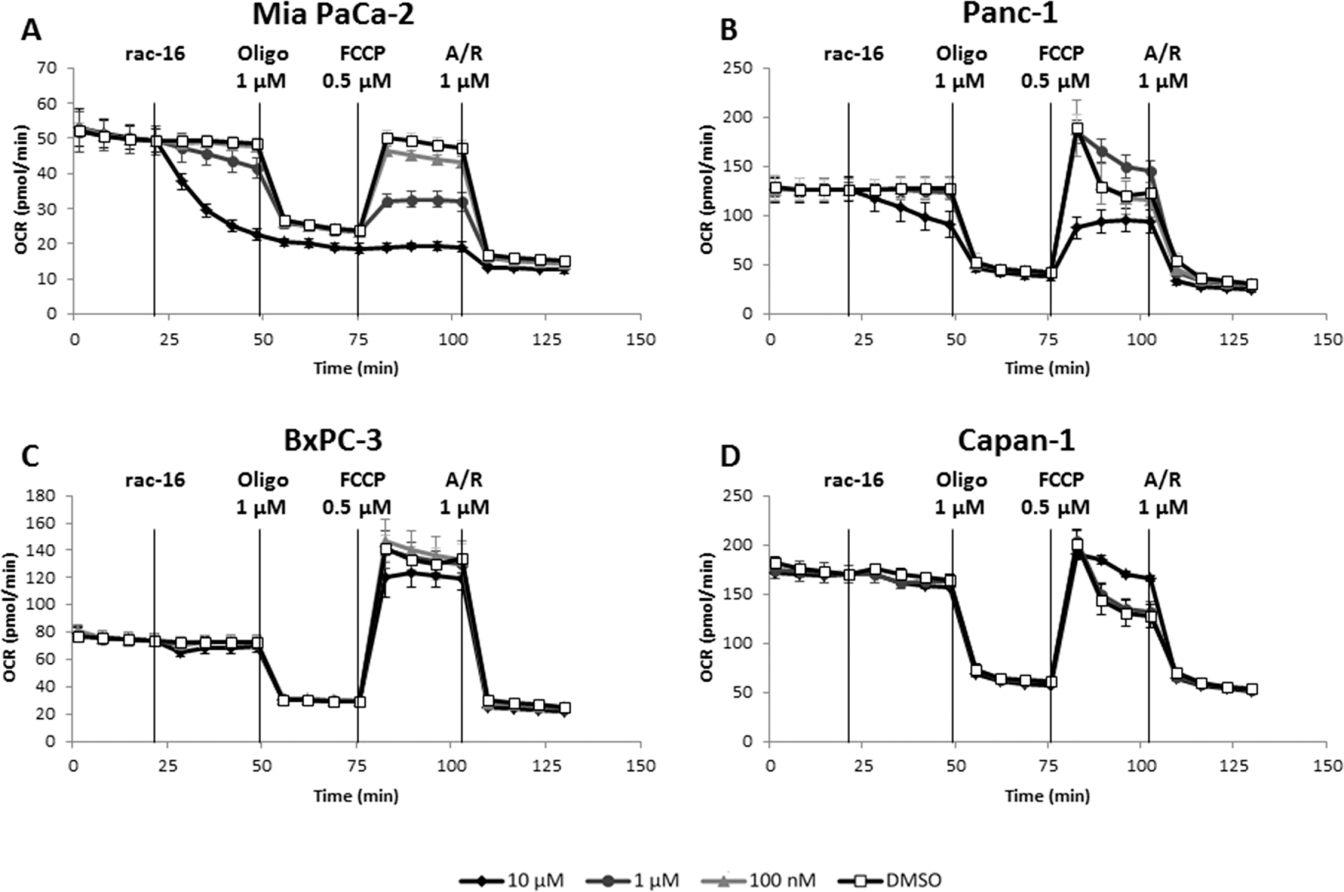
K_Ca_3.1 inhibition modulates oxygen consumption profile in a subset of PDAC cell lines. **(A)** Seahorse XF Mito Stress test was performed to measure mitochondrial function upon various concentrations of rac-16 treatment of Mia PaCa-2, **(B)**, Panc-1, **(C)** BxPC-3, and **(D)** Capan-1 cells. n = 3. Oligo is oligomycin A, FCCP is carbonyl cyanide-4-(trifluoromethoxy)phenylhydrazone, A/R is a mix of antimycin A and rotenone. n = 6.

To further characterize the effect of K_Ca_3.1 channel inhibition, we performed the Seahorse XFMito Stress test to analyze the influence of rac-16 on maximal expiration (Fig 4). As described before, adding rac-16 lead to a dose-dependent decrease of basal respiration in MiaPaCa-2 cells. As expected, the complex V inhibitor oligomycin completely abolished mitochondrial respiration in the presence or absence of rac-16. Interestingly, after the addition of the uncoupling agent FCCP, also maximal respiration is strongly inhibited by rac-16 in a dose-dependent manner in Mia PaCa-2 cells, demonstrating that K_Ca_3.1 channel also regulates respiration under conditions of mitochondrial stress. Maximal respiration of Panc-1 cells was inhibited only at 10 μM, only weakly inhibited in BxPC-3 cells and not affected in Capan-1 cells.

To assess the specificity of the observed inhibitor effects, we compared the effect of rac-16 on cells treated with K_Ca_3.1 channel siRNA versus control siRNA (Figure B in S1 File). As described before, K_Ca_3.1 channel siRNA leads to a significant inhibition of OCR compared to controls. Subsequent treatment with rac-16 has, most likely due to residual channel expression after K_Ca_3.1 channel knock-down, smaller effects on K_Ca_3.1 channel siRNA treated cells than on control cells. The final OCR is comparable between K_Ca_3.1 channel siRNA and control cells after rac-16 treatment. These results confirm the specificity of the effect of rac-16 on K_Ca_3.1.

### K_Ca_3.1 channel inhibition affects OxPhos-dependent cell proliferation

**Fig 5:**
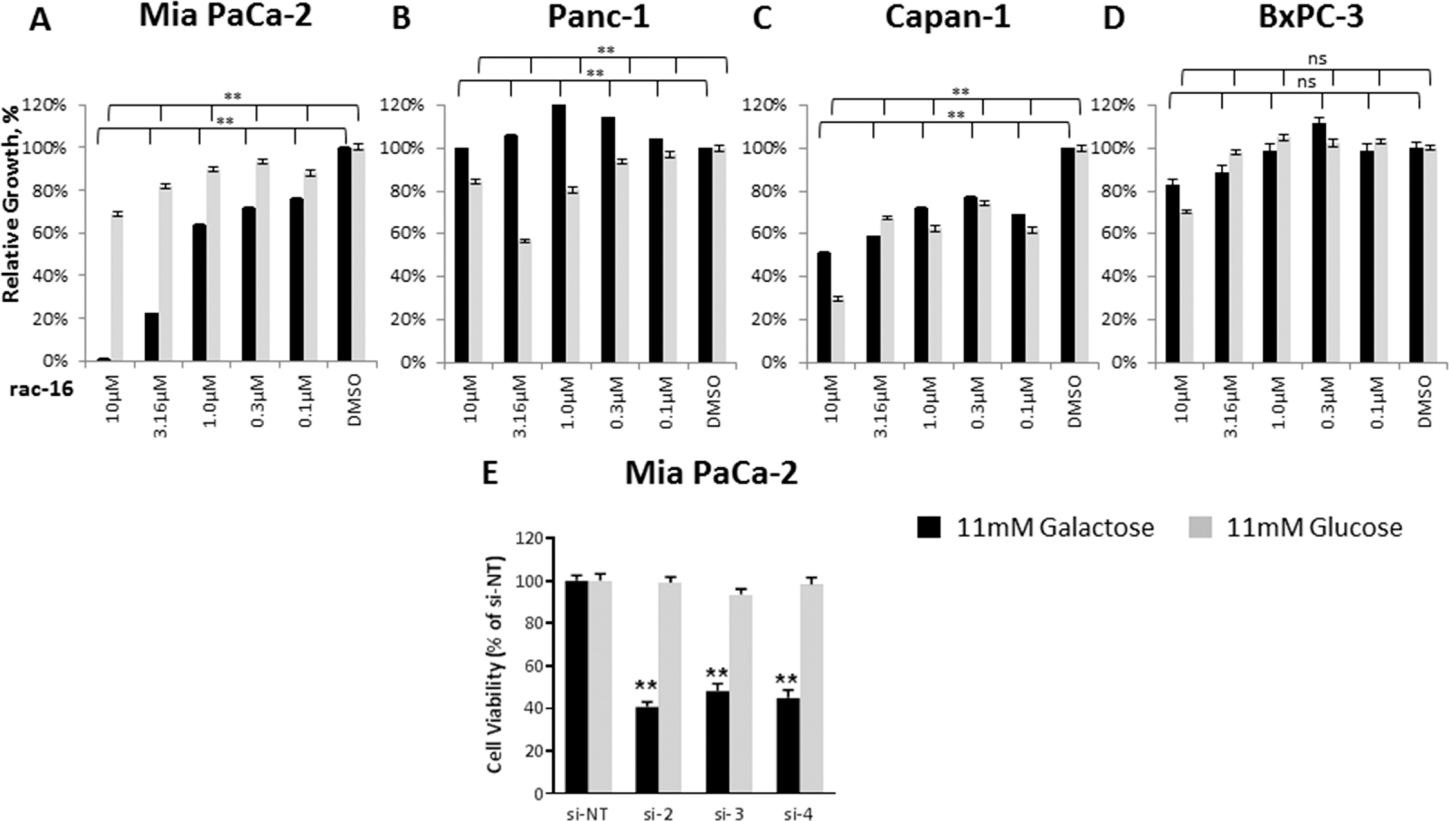
K_Ca_3.1 inhibition affects proliferation. **(A)** xCELLigence proliferation assay on Mia PaCa-2, **(B)** Panc-1 **(C)** Capan-1 and **(D)** BxPC-3 cells grown in the presence of 11 mM glucose or galactose as the main energy source and treated with increasing concentrations of rac-16, n = 5 for each condition. The slope of the exponential phase of the growth curve was calculated in the complementary software. The relative growth was calculated as the ratio between the slopes of DMSO-and rac-16 treated cells in the appropriate media. **(E)** Viability assay of Mia PaCa-2 cells transfected with non-targeted siRNA or individual siRNAs against KCNN4. Assay was conducted 72 hours post-transfection, n = 3. ANOVA Bonferroni test was used to compare each of the rac-16 treatments vs DMSO; ns, not significant, p>0.05,*, p-value is 0.01 to 0.05; **, p-value < 0.01.

To determine the effect of K_Ca_3.1 channel inhibition on proliferation of PDAC cell lines, Mia PaCa-2, Panc-1, Capan-1 and BxPC-3 cells were incubated with increasing concentrations of rac-16 (Fig 5A–5D). Replacing 11 mM glucose with 11 mM galactose to shift cellular metabolism towards OxPhos allowed determining effects on OxPhos by measuring cell proliferation. In all cell lines tested, inhibition of K_Ca_3.1 channel had either no effect on proliferation or similar, non dose-dependent effects on cell proliferation in the presence of either carbon source. However, only Mia PaCa-2 cells showed a dose-dependent galactose-specific effect after rac-16 administration. To further confirm these results and the specificity of the inhibitor, Mia PaCa-2 cells were transfected with three independent siRNAs (si-2, 3 and 4) and replated to assess cell proliferation. As observed for rac-16, knock-down of KCNN4 by siRNA had no effect on cell proliferation of Mia PaCa-2 cells in the presence of 11 mM glucose, but led to significantly reduced cell number in the presence of 11 mM galactose (Fig 5E) 72 hours post-transfection. This indicates a correlation between the effect of the inhibitor on oxygen consumption and tumor cell proliferation in galactose.

### Mia PaCa-2 cells express a mitochondrial form of KCNN4

**Fig 6:**
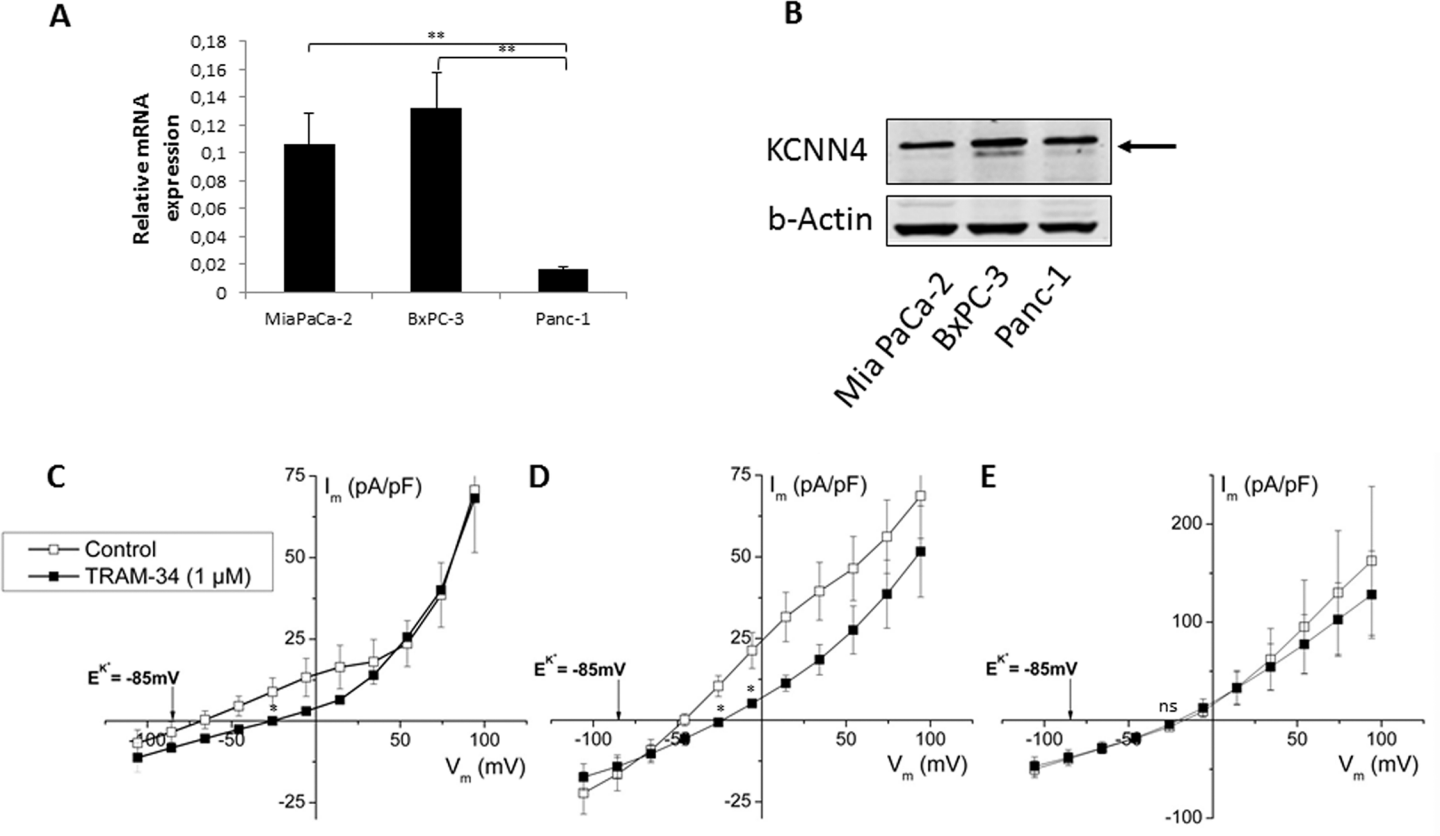
K_Ca_3.1 expression and function in the panel of PDAC cell lines. **(A)** *KCNN4* TaqMan gene expression assay, KCNN4 mRNA levels were normalized to *HPRT* **(B)** and Western Blot analysis of KCNN4 protein expression, β-Actin was used as a loading control in PDAC cell lines. Arrow indicates the KCNN4 protein band **(C)** Whole-cell patch-clamp recordings in the presence of 1 μM free Ca^2+^ in pipette solution in Mia PaCa-2, n = 7; **(D)** BxPC-3, n = 11; **(E)** Panc-1 cells, n = 7. TRAM-34 was used as a specific K_Ca_3.1 inhibitor. Tukey’s Multiple Comparison ANOVA Test was performed. p>0.05,*, p-value is 0.01 to 0.05; **, p-value < 0.01.; for the patch-clamp significance was calculated at the measurement point of KCNN4 current using ANOVA t-test, ns, not significant, p>0.05, *, p-value is 0.01 to 0.05.

K_Ca_3.1 channels are described to be expressed in the plasma membrane as well as in the mitochondria [30, 31]. To investigate the underlying mechanisms of K_Ca_3.1 channel mediated regulation of oxygen consumption, K_Ca_3.1 channel mRNA and protein levels as well as K_Ca_3.1 channel activity was determined. The K_Ca_3.1 channel inhibitor-sensitive cell line Mia PaCa-2 expresses K_Ca_3.1 channel on mRNA and protein level (Fig 6A and 6B). To confirm functional expression of the channel in the plasma membrane, we performed patch-clamp measurements in the whole cell configuration and in presence of 1 μM free Ca^2+^ in pipette solution. Both BxPC-3 and Mia PaCa-2 cells exhibited current-voltage relationships that revert close to the calculated reversal potential of K^+^ (E_K_^+^ = -85 mV), show inward rectification at positive potential and were sensitive to the K_Ca_3.1 channel -specific inhibitor TRAM-34. All these are characteristics as described for the K_Ca_3.1 channel [32, 33]. These currents were not detected in Panc-1 cells. (Fig 6C–6E, Figure C in S1 File).

**Fig 7:**
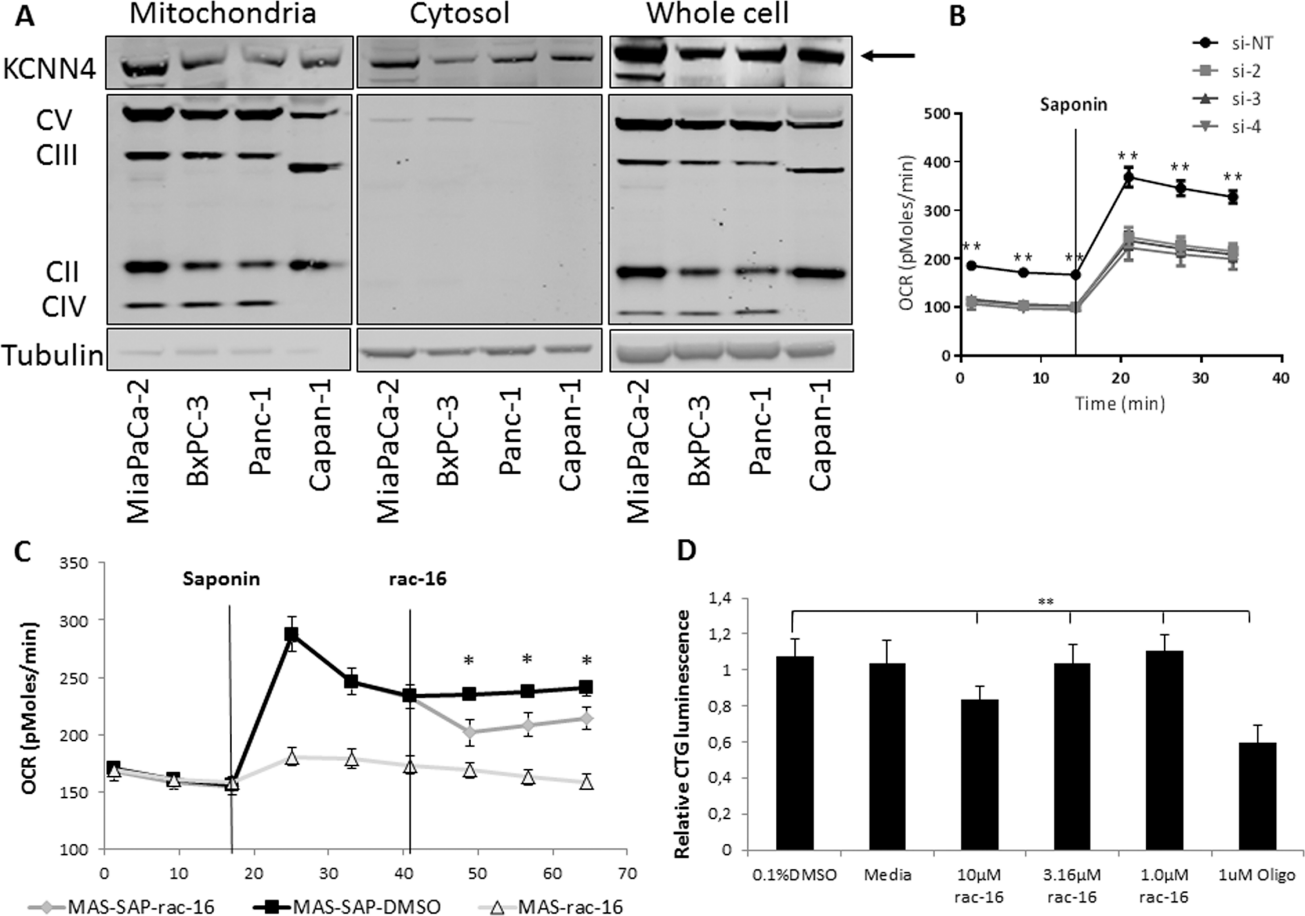
MitoK_Ca_3.1 regulates mitochondrial respiration in Mia PaCa-2 cells. **(A)** Western Blot analyses of Mia PaCa-2, BxPC-3, Panc-1 and Capan-1 cells; mitochondrial fraction was prepared from equal cell number of each cell line, supernatant from mitochondrial fraction preparation was taken as the cytosolic fraction. Fractions were compared among each other and to whole cell protein lysates. Antibodies recognizing mitochondrial complex V, III, II, IV were used as mitochondrial controls and tubulin as the cytosolic control. Arrow indicates the KCNN4 protein band. **(B)** OCR measurement of permeabilized in Mannitol and Sucrose buffer (MAS) by 25 μg/ml saponin (SAP) Mia PaCa-2 cells transfected with individual siRNAs against KCNN4, 30 000 cells/well were re-seeded the night before the assay to avoid cell number-dependent changes, n = 4 for each condition, p<0.001. **(C)** OCR measurement of permeabilized in Mannitol and Sucrose buffer (MAS) by 25 μg/ml saponin (SAP) Mia PaCa-2 cells treated with 10 μM K_Ca_3.1 inhibitor rac-16, n = 32, p<0.01; **(D)** Mitochondrial ATP production in Mia PaCa-2 cells upon treatment with varying concentrations of the K_Ca_3.1 inhibitor rac-16 or DMSO, 1 μM Oligomycin (Oligo) served as a positive control, n = 6, p<0.0001. Bonferroni ANOVA test was performed to compare Non-Targeting siRNA vs siRNA targeting KCNN4 mRNA or DMSO-vs rac-16 treatment; ns, not significant, p>0.05, *, p-value is 0.01 to 0.05; **, p-value < 0.01.

These results show a correlation between the presence of functional K_Ca_3.1 channel in the plasma membrane and the effect of K_Ca_3.1 channel inhibition on cellular oxygen consumption and proliferation, indicating a possible role of K_Ca_3.1 channel expressed on the cell surface. However, K_Ca_3.1 channel was also described to be expressed in the mitochondria and mitochondrial expression of the channel indicates an involvement in metabolic processes. Therefore, the mitochondrial expression of K_Ca_3.1 channel was analyzed in Mia PaCa-2, Panc-1 Capan-1 and BxPC-3 cells. Western Blot analyses of mitochondrial extracts showed highest K_Ca_3.1 channel protein expression in Mia PaCa-2 cells (Fig 7A). To assess if this mitochondrial K_Ca_3.1 channel isoform is involved in the regulation of cellular oxygen consumption, metabolic analyses were performed in permeabilized Mia PaCa-2 cells to exclude the role of cell surface expressed K_Ca_3.1 channel. Mia Paca-2 cells were transfected with three independent siRNAs (si-2, 3 and 4) to knock-down KCNN4 and assessed for OCR before and after permeabilization of the outer cell membrane. As described before, knock-down of KCNN4 led to a significant decrease of OCR in intact MiaPaCa-2 cells. Permeabilization of the outer membrane induced a drastic increase in OCR; however, the difference in OCR between KCNN4 knock-down cells and control transfected cells remained constant before and after permeabilization indicating a role of the mitochondrial protein in the regulation of OCR (Fig 7B). Furthermore, incubation of permeabilized cells with 10 μM rac-16 led to a significant decrease in oxygen consumption, again indicating a role of mitochondrial K_Ca_3.1 channel in the regulation of this process (Fig 7C). Finally, the influence of K_Ca_3.1 channel inhibition on OxPhos-dependent ATP generation was assessed. Mia PaCa-2 cells were incubated for 4 hours with increasing concentrations of rac-16 in the presence of galactose as the sole carbon source. Inhibition of K_Ca_3.1 channel by rac-16 resulted in a 20% decrease in ATP production compared to DMSO-treated control or a 50% depletion of ATP compared to the inhibitor of cellular respiration oligomycin, indicating a role of mitochondrial K_Ca_3.1 channel in the functionality of mitochondrial respiration and ATP production (Fig 7D).

## Discussion

The study presented here aimed to establish an unbiased siRNA screening approach to identify modulators of OxPhos among ion channels and transporters in PDAC cells. Although the siRNA-based screen identified many hits that were previously described to regulate metabolism, and thereby confirms the validity of this study, the screen nonetheless has a number of limitations. One of the major limitations being the lack of cellular heterogeneity present within and between different tumors [34], [35], [36], [37]. The screen focused on Mia PaCa-2 cells as these cells displayed sufficient oxidative capacity to perform the screen and were reliably and potently transfectable by a wide variety of different siRNAs. The use of additional pancreatic cancer cell lines or of different tumor entities would allow for the characterization of the metabolic differences between different cancer subtypes. Additionally, most solid tumors have varied niches characterized by differences in the supply of nutrients and oxygen [38]. Low oxygen, resulting in the upregulation of hypoxia-inducible factors (HIFs), is described to be an important regulator of cellular metabolism by inducing the expression of glucose transporters, glycolytic and other metabolic enzymes [39], [40]. Future screens under different environmental conditions could potentially identify additional regulators of tumor metabolism.

A detailed analysis of screened genes revealed a subset of known modulators of metabolism which did not appear as regulators of metabolism in the present approach (e.g., *MCT1*, *SLC25A10*). This could be due to low expression of target mRNAs in Mia PaCa-2 cells, the presence of compensatory mechanisms, insufficient transfection efficiency, inappropriate timing for phenotypic readout, or a limited sensitivity of the assay read out. Nevertheless, we were able to confirm screening hits by using additional individual siRNAs, as well as small molecule inhibitor and thereby showing the validity of our approach.

We identified several proteins, including K_Ca_3.1 channels as the number one hit, previously not associated with the regulation of tumor metabolism. We recorded differential functional expression of K_Ca_3.1 channel in the panel of PDAC cell lines which correlates with a previously published pattern [41], however does not correlate with the influence of the rac-16 K_Ca_3.1 inhibitor on respiration. Additionally K_Ca_3.1 channel was found to be expressed in the mitochondria of these cells. Seahorse Analyzer XF experiments on permeabilized cells show that the mitochondrial expressed K_Ca_3.1 channel is at least partially responsible for the observed phenotype. However, based on our results an additional role of cell surface expressed K_Ca_3.1 channel on the regulation of cellular oxygen consumption cannot be excluded.

K_Ca_3.1 channels are expressed in erythrocytes, lymphocytes, liver and pancreas as well as in vascular smooth muscle, endothelial and blood cells [42]. They are important regulators of vasorelaxation and smooth muscle cell regulation [43], [44]. In addition, K_Ca_3.1 channels were shown to be expressed in different cancer cell lines and inhibition has been shown to attenuate neoplastic cell growth both in vitro and in vivo [45], [46], [47], [48]. K_Ca_3.1 channels were shown to be overexpressed in 32% of glioma patients and the expression correlates with poor prognosis in both glioma and NSCLC patients [47, 49]. K_Ca_3.1 channels have also been characterized as an important driving force for anion extrusion in normal pancreatic ducts and their roles in normal physiology, overexpression on PDAC [41], and role in pancreatic pathophysiology were described [50] [51].

Finally, the subcellular localization of K_Ca_3.1 channels was shown to be crucial for the regulation of cell migration [52]. Mitochondrial expression of K_Ca_3.1 channels has been described previously in HCT116 cells [30]. De Marchi and colleagues found K_Ca_3.1 channels in the inner mitochondrial membrane where it is regulated by small changes of the mitochondrial matrix Ca^2+^ concentration. Here, we describe for the first time a role for K_Ca_3.1 channels in the regulation of oxygen consumption. We were able to confirm mitochondrial expression of K_Ca_3.1 channels in a subset of PDAC cell lines and we suggest that its mitochondrial expression correlates with the regulation of oxygen consumption. Furthermore, we have shown mitochondrial expressed K_Ca_3.1 channels to be at least partially responsible for the effects of K_Ca_3.1 channels inhibitor on oxygen consumption. Although a role of mitochondrial expressed K_Ca_3.1 channels in the regulation of oxygen consumption seems suggestive, we cannot exclude an additional influence of K_Ca_3.1 channels in the plasma membrane.

Sassi and colleagues did not observe any influence of mitochondrial K_Ca_3.1 channels on cell proliferation using TRAM-34 as an inhibitor of K_Ca_3.1 channels [31]. As TRAM-34 is generally known to have poor solubility and high protein binding, we used the previously described K_Ca_3.1 channel inhibitor rac-16. Our study confirms the findings of Sassi et al. in the presence of sufficient glucose. However, forcing cells to generate ATP exclusively via OxPhos using galactose media sensitizes tumor cells expressing mitochondrial K_Ca_3.1 channels to corresponding inhibitors and reduces proliferation of these cells. The small molecule inhibitor rac-16 was described to inhibit K_Ca_3.1 channels in a dose-dependent manner with an IC_50_ of 8 nM [26]. In contrast, significant higher concentrations of rac-16 are needed to inhibit OxPhos in Mia PaCa-2 cells. However, the IC_50_ of rac-16 was originally determined by measuring ionomycin-induced Rb^+^ efflux of preloaded C6BU1 cells and depends very much on channel and substrate concentrations in the experimental settings. In addition, mitochondrial channels tend to be less sensitive to inhibitors compared to their plasma-membrane counterparts as a portion of the drug is likely to be retained in cellular membranes [53]. Off-target effects of rac-16 cannot be excluded at higher concentrations. However, rac-16 exerts no effects against a panel of GPCRs and ion channels up to 10μM (data not shown). Finally, different isoforms of KCNN4 are described to differ in their sensitivity to K_Ca_3.1 channel inhibitors [54]. Therefore, we can also not exclude the possibility of a specific isoform or modification of the protein that changes sensitivity towards inhibitors.

The exact mechanisms of K_Ca_3.1 channels mediated control of metabolism and its functional implications have to be elucidated in future studies. However, previous reports have described a role of potassium ions in the regulation of the mitochondrial membrane potential, which is a driving force for the respiratory chain [53]. Additionally, K^+^-flux was described to regulate mitochondrial volume and as a consequence to regulate respiration. [55] Therefore, modulating ion concentrations will influence the activity of the respiratory chain and thereby, ATP production and oxygen consumption.

## Supporting Information

S1 File **Figure A. KCNN4 and extracellular acidification. (A)** Extracellular Acidification Rate measurements of Mia PaCa-2, **(B)** Panc-1, **(C)** Capan-1 and (D) BxPC-3 cells treated with rac-16 (KCNN4 inhibitor) at different concentrations as indicated, 100 nM NS309 (KCNN4 activator) and high concentrations of extracellular KCl (60 mM), n = 5. Data are represented as the mean ± SD. **Figure B. KCNN4 hit confirmation.** Mia PaCa-2 cells transfected with siRNA against KCNN4, 20 000 cells/well were re-seeded one the night before the assay, upon 6 baseline measurements 10 μM of rac-16 or 0.1% DMSO final concentration were injected to the media, n = 6. **Figure C. Differential amplitude of TRAM-34 sensitive current in the panel of PDAC cell lines.** Analyses of the whole-cell patch-clamp recordings in the presence of 1 μM free Ca2+ in pipette solution at -5.8 mV potential in Mia PaCa-2 (n = 7), BxPC-3 (n = 11) and Panc-1 cells (n = 7).
(PPTX)

S1 Table List of transportome genes used for the screening on Mia PaCa-2 cells. (XLSX)
