# Supplementary material for: Identification of KCa3.1 Channel as a Novel Regulator of Oxidative Phosphorylation in a Subset of Pancreatic Carcinoma Cell Lines
Source: PLoS One. 2016 Aug 5;11(8):e0160658. doi: 10.1371/journal.pone.0160658 (PMC4975431; doi:10.1371/journal.pone.0160658)
Supplement: S1 File — Figure A. KCNN4 and extracellular acidification. (A) Extracellular Acidification Rate measurements of Mia PaCa-2, (B) Panc-1, (C) Capan-1 and (D) BxPC-3 cells treated with rac-16 (KCNN4 inhibitor) at different concentrations as indicated, 100 nM NS309 (KCNN4 activator) and high concentrations of extracellular KCl (60 mM), n = 5. Data are represented as the mean ± SD. Figure B. KCNN4 hit confirmation. Mia PaCa-2 cells transfected with siRNA against KCNN4, 20 000 cells/well were re-seeded one the night before the assay, upon 6 baseline measurements 10 μM of rac-16 or 0.1% DMSO final concentration were injected to the media, n = 6. Figure C. Differential amplitude of TRAM-34 sensitive current in the panel of PDAC cell lines. Analyses of the whole-cell patch-clamp recordings in the presence of 1 μM free Ca2+ in pipette solution at -5.8 mV potential in Mia PaCa-2 (n = 7), BxPC-3 (n = 11) and Panc-1 cells (n = 7). (PPTX) [file pone.0160658.s001.pptx]

## Slide 1
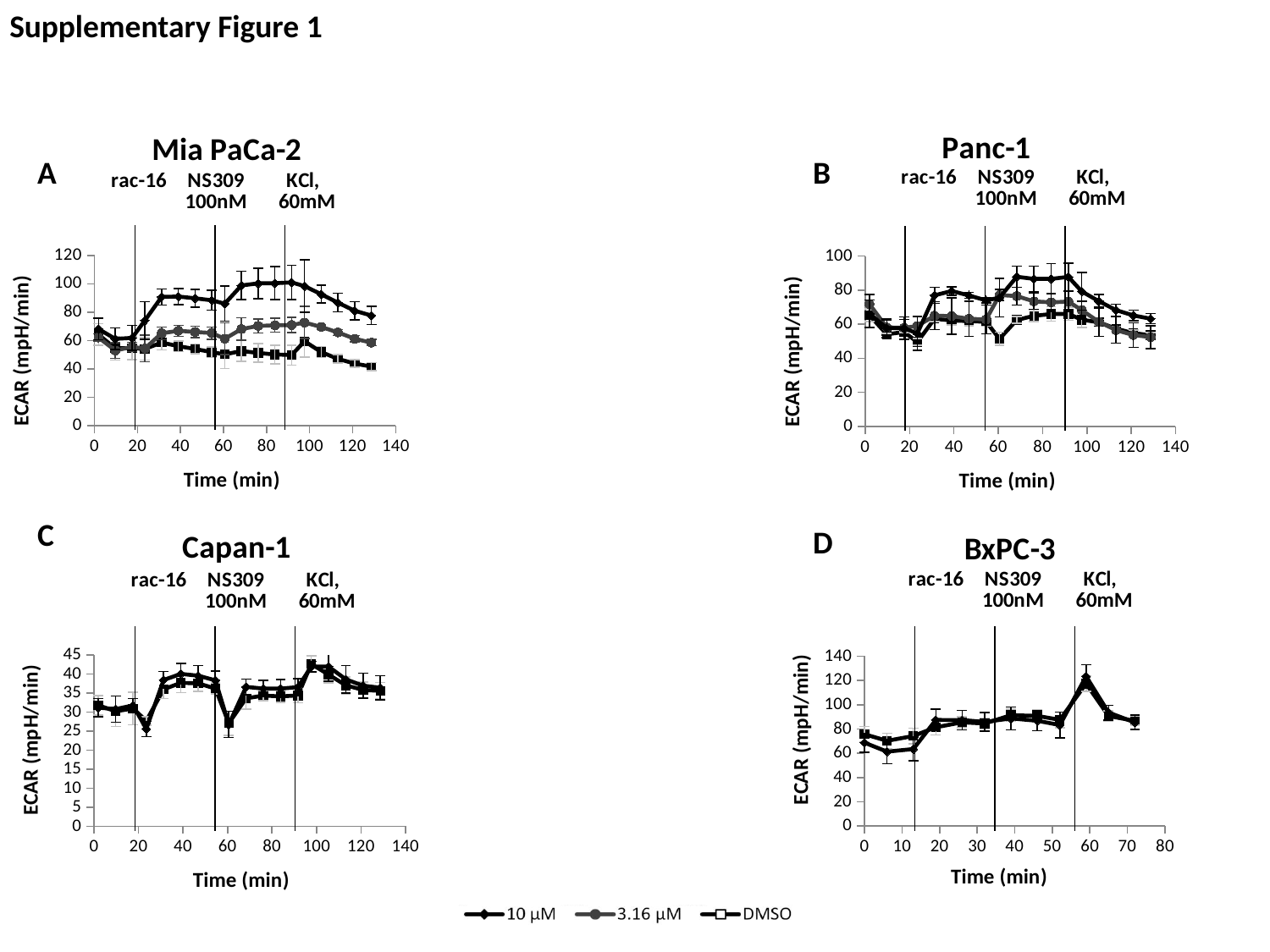

Supplementary Figure 1
### Chart:
| Category | 10 μM | 3.16 μM | DMSO |
|---|---|---|---|
### Chart: Mia PaCa-2
| Category | 10 μM | 3.16 μM | DMSO |
|---|---|---|---|A
B
### Chart:
| Category | 10 μM | DMSO |
|---|---|---|
### Chart: BxPC-3
| Category | 10 μM | DMSO |
|---|---|---|D

## Slide 2
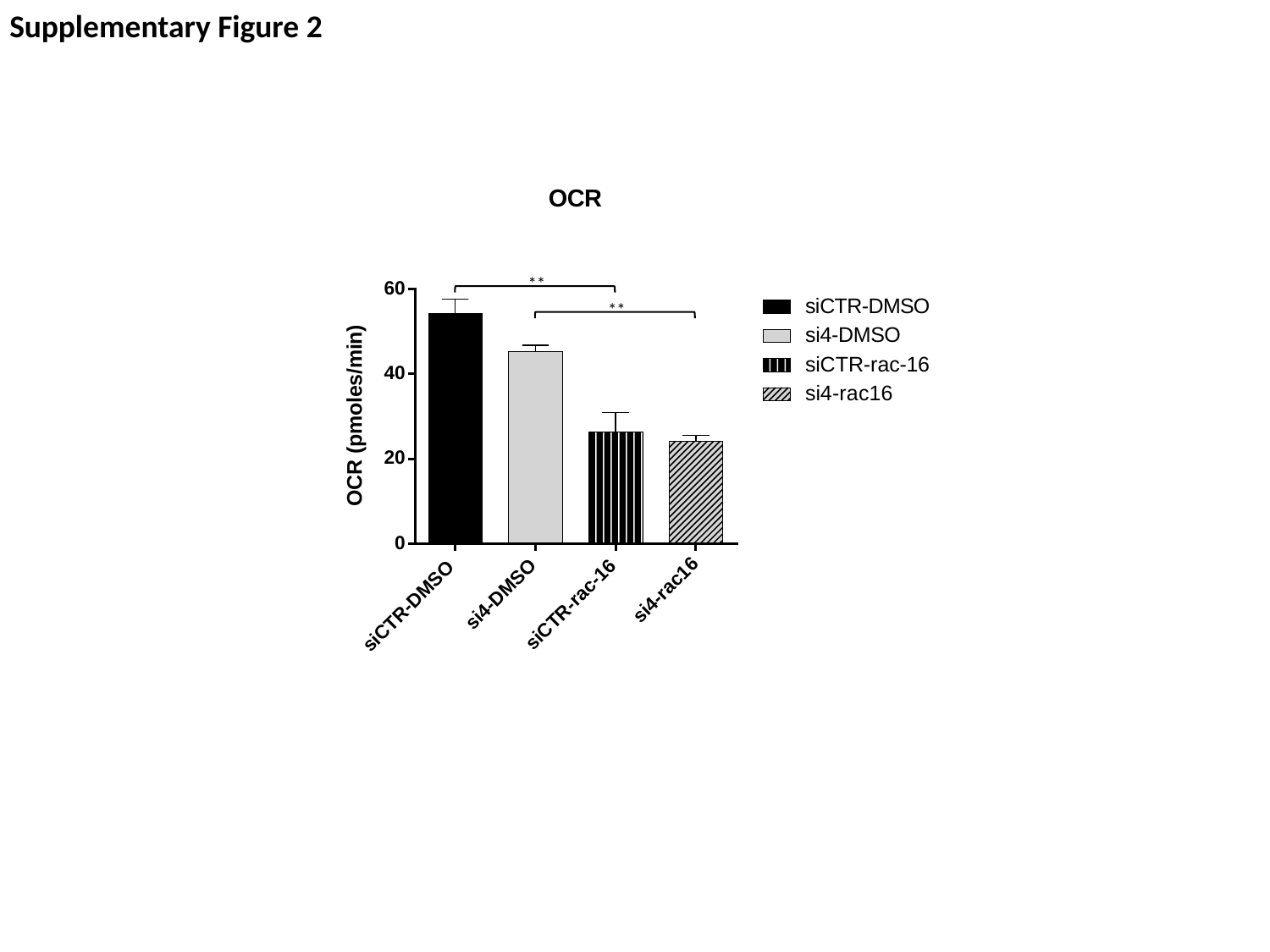

Supplementary Figure 2
**
**

## Slide 3
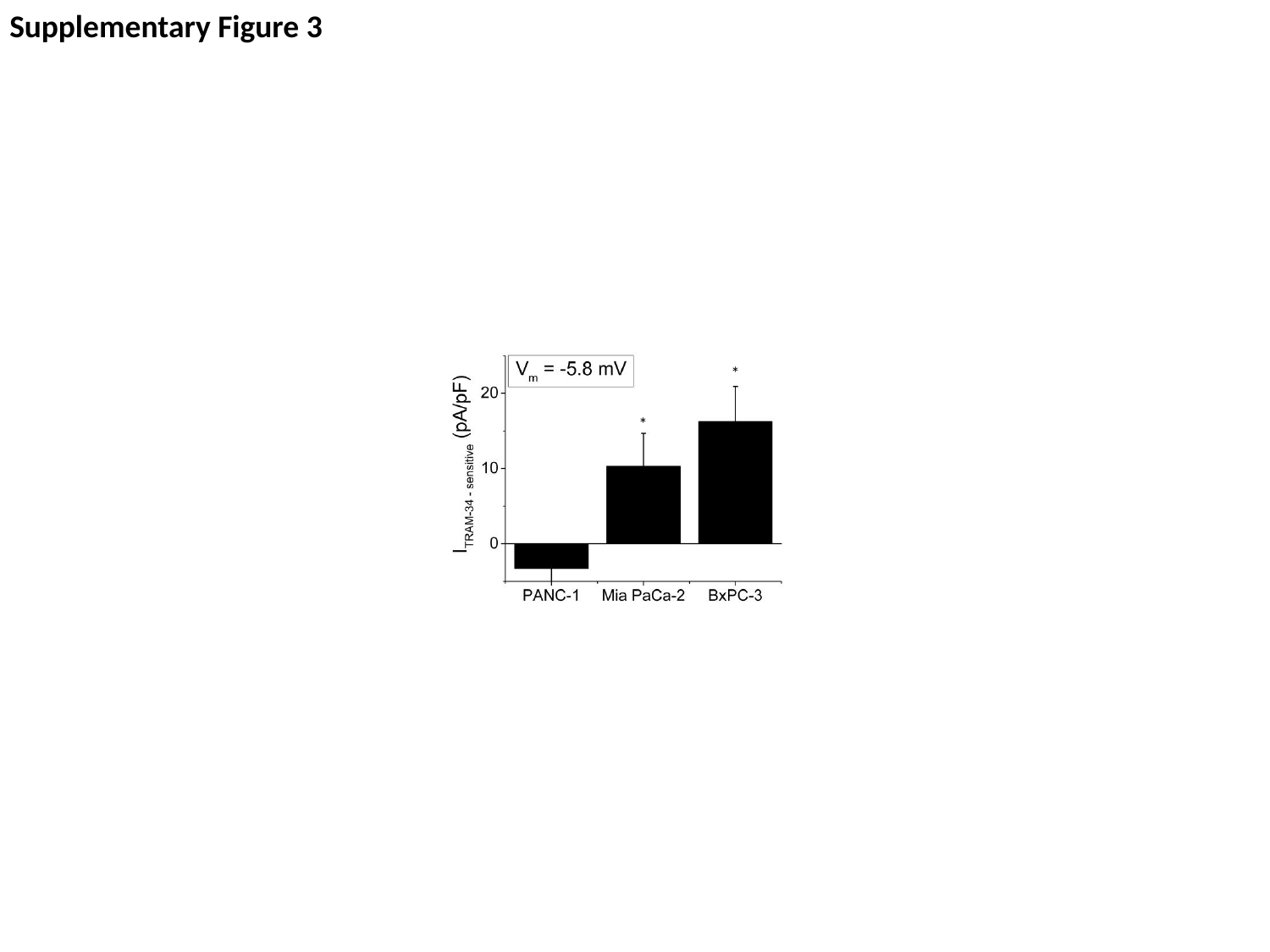

Supplementary Figure 3
*
*
